# A comparison of frailty measures in population-based data for patients with colorectal cancer

**DOI:** 10.1093/ageing/afae105

**Published:** 2024-05-23

**Authors:** Rebecca Birch, John Taylor, Tameera Rahman, Riccardo Audisio, Sophie Pilleron, Philip Quirke, Simon Howell, Amy Downing, Eva Morris

**Affiliations:** Leeds Institute for Medical Research at St James’s, University of Leeds, Leeds, UK; Leeds Institute for Medical Research at St James’s, University of Leeds, Leeds, UK; Health Data Insight CIC, Cambridge, UK; National Disease Registration Service, NHS England, London, UK; Department of Surgery, Sahlgrenska University Hospital, Gothenburg, Sweden; Ageing, Cancer, and Disparities Research Unit, Department of Precision Health, Luxembourg Institute of Health, 1A-B, rue Thomas Edison, 1445 Strassen, Luxembourg; Leeds Institute for Medical Research at St James’s, University of Leeds, Leeds, UK; Leeds Institute for Medical Research at St James’s, University of Leeds, Leeds, UK; Leeds Teaching Hospitals NHS Trust, Leeds, UK; Leeds Institute for Medical Research at St James’s, University of Leeds, Leeds, UK; Nuffield Department of Population Health, Big Data Institute, University of Oxford, Oxford, UK

**Keywords:** colorectal cancer, frailty, inequalities, survival, inequities, older people

## Abstract

**Background:**

Numerous studies have revealed age-related inequalities in colorectal cancer care. Increasing levels of frailty in an ageing population may be contributing to this, but quantifying frailty in population-based studies is challenging.

**Objective:**

To assess the feasibility, validity and reliability of the Hospital Frailty Risk Score (HFRS), the Secondary Care Administrative Records Frailty (SCARF) index and the frailty syndromes (FS) measures in a national colorectal cancer cohort.

**Design:**

Retrospective population-based study using 136,008 patients with colorectal cancer treated within the English National Health Service.

**Methods:**

Each measure was generated in the dataset to assess their feasibility. The diagnostic codes used in each measure were compared with those in the Charlson Comorbidity Index (CCI). Validity was assessed using the prevalence of frailty and relationship with 1-year survival. The Brier score and the c-statistic were used to assess performance and discriminative ability of models with included each measure.

**Results:**

All measures demonstrated feasibility, validity and reliability. Diagnostic codes used in SCARF and CCI have considerable overlap. Prevalence of frailty determined by each differed; SCARF allocating 55.4% of the population to the lowest risk group compared with 85.1% (HFRS) and 81.2% (FS). HFRS and FS demonstrated the greatest difference in 1-year overall survival between those with the lowest and highest measured levels of frailty. Differences in model performance were marginal.

**Conclusions:**

HFRS, SCARF and FS all have value in quantifying frailty in routine administrative health care datasets. The most suitable measure will depend on the context and requirements of each individual epidemiological study.

## Key Points

- The three measures of frailty included classified differing proportions of the colorectal cancer population as frail.
- The prevalence of frailty increased with chronological age in all three measures.
- Higher levels of measured frailty were associated with higher rates of death within a year of colorectal cancer diagnosis.
- All three frailty measures included were able to quantify frailty and could be used more widely in epidemiological studies.

## Introduction

Frailty is a concept which describes declines in multiple physiological systems, resulting in reduced resistance to stressors and increases risk of adverse clinical outcomes [1, 2]. As people age their risk of becoming frail increases [3], so does their risk of developing cancer [4]. Those who experience both face significant challenges, as frailty may limit the ability to receive potentially curative treatments, extends recovery times and increases the risk of morbidity and mortality [5–7].

Colorectal cancer (CRC) is a common cancer affecting over 42,000 people each year in the UK, over 40% of these will be over the age of 75 at diagnosis [8]. UK outcomes from CRC lag behind those attained by many comparable countries and the deficit is driven by poor outcomes amongst older people [9]. Drivers of these age-related inequities and inequalities are unclear [10, 11] and the contribution frailty makes is not well understood. Given its potential influence on management and outcome [7, 12], the ability to quantify frailty in population-level data, and incorporate it into epidemiological studies, is vital. To date, however, it has not been possible. As a result, many such studies are limited by simply considering age in their investigations of variation in care [13, 14] which, given older adults are a heterogeneous group in terms of health and fitness level, is inadequate.

Frailty is related to co-morbidity which has, to date, been more routinely considered. Co-morbidity can be defined as the simultaneous presence of two or more medical conditions and, like frailty, its incidence increases with age. Whilst frailty and co-morbidity are closely related they are different concepts and, ideally, should be considered separately [1, 15]. Particularly as studies have shown that the presence of increasing levels of co-morbidity alone do not account for the poor outcomes observed in older patients [16].

In the clinical setting there are a number of assessments that can be used to make an objective and robust quantification of frailty [2, 17–23]. Quantifying frailty in population-based administrative datasets that are used for epidemiological studies is more challenging. Several frailty measures have been developed for use in these datasets, but their relative strengths and weaknesses have not been explored in comparison with each other [24–26]. Given the scale of age-related inequalities and inequities in CRC care, there is an urgent need to better quantify frailty and assess its impact [3, 5, 7, 10, 11]. This study seeks to compare the utility of three commonly utilised indices of frailty which can be derived and applied in HES data in England: the Hospital Frailty Risk Score (HFRS) [24], Secondary Care Administrative Records Frailty (SCARF) index [25] and a frailty syndromes (FS) model [26].

## Materials and methods

### Data and study population

The utility of these measures was investigated in a national cohort of patients with CRC. This consisted of all individuals aged ≥18, diagnosed with the disease (International Classification of Diseases Version 10 (ICD10) codes [27] C18-C20) in England between 2016 and 2019. Linked cancer registration and Hospital Episode Statistics (HES) [28] data were obtained from the COloRECTal cancer data Repository (CORECT-R) [29]. Information used included age group (18–64, 65–74, 75–84 and ≥85 years), sex, tumour site (colon or rectum), tumour stage (I to IV or unknown) and survival time (in days calculated from date of diagnosis to date of death or censoring on 31 December 2020). Information was provided on all diagnostic codes included in the different frailty indices at attendances at National Health Service (NHS) hospitals derived from HES data in the 2 years prior to diagnosis.

### Overview of frailty measures

Three frailty measures were investigated, all of which were created from, and designed for use in HES, an English hospital discharge dataset in which all diagnostic reasons for daycase and inpatient admissions are coded using ICD10 codes. Primary care data were not included in this project precluding those measures constructed from such data.

The first measure utilised was the HFRS [24]. This was developed from a HES dataset containing admissions of patients, with and without cancer, aged 75 years and over. ICD10 codes were given a weighted score proportional to the strength of prediction for hospital outcomes. The HFRS score is calculated by summing the score over all 109 codes for each individual. The categorisation of the HFRS for an individual was based on the maximum discrimination of the outcomes: low risk (HFRS <5), moderate risk (HFRS 5–15) and high risk (HFRS >15). In the development cohort utilised by Gilbert et al., 66.0% of individuals were classified as low risk, 20.3% as moderate and 13.7% as high risk [24].

The second was the SCARF index [25]. This was developed using a national cohort of women aged 50 years and over with oestrogen receptor positive early invasive breast cancer. This index includes codes for 32 health and function deficits. The SCARF index for each individual is calculated as the total number of deficits divided by the total in the index (*n* = 32). The following categorisation of the index is recommended: fit (0–1 deficit, index ≤0.05); mild frailty (2–3 deficits, index 0.06–0.11); moderate frailty (4–5 deficits, index 0.12–0.18); severe frailty (≥6 deficits, index ≥0.19).

The final measure was a risk prediction model for acute care [26], not restricted to individuals with cancer, based on a national cohort of individuals aged ≥65 years admitted as an emergency [26]. The authors classified patients across the following FS: anxiety and depression, cognitive impairment (including senility, dementia and delirium), functional dependence, falls and fractures, incontinence, mobility problems and pressure ulcers. To quantify the prevalence of frailty, the authors also used a count of the FS [30], which has been employed in this study (0 frailty syndromes, 1 frailty syndrome, 2 frailty syndromes and ≥3 frailty syndromes).

### Statistical analysis

The three frailty measures were derived for each person in the cohort. The characteristics of the study population were assessed, both overall and in relation to their vital status (dead/alive) at 1 year from diagnosis. Due to the known relationship between age and frailty and the fact the measures were developed in different age profiles, analyses were repeated stratified by age (<75 & ≥75). This split was selected as 74 marks the end of the eligibility for routine CRC screening within the English NHS and this group has been shown to be the most rapidly growing proportion of the population in England [31].

To investigate the utility of the three frailty measures, their feasibility, validity and reliability were assessed following an approach previously used to compare co-morbidity measures [32]. The proportion of each population for which an indication of frailty could be determined was calculated in order to assess feasibility.

To assess the content and face validity, the prevalence of frailty as assessed by each measure across the whole cohort and by year of age at cancer diagnosis was assessed. As frailty has a strong inverse relationship with survival, 1-year Kaplan–Meier curves were estimated for each measure, both overall and stratified by age (<75 and ≥75). The log-rank test was used to test the equality of survivor functions across categorical levels of frailty for each measure.

Predictive validity was assessed by fitting a baseline logistic regression model with 1-year mortality as the outcome and age, sex, tumour site and tumour stage at diagnosis as predictors. These predictors were selected due to their known relationship with survival from CRC [33–38]. Each measure of frailty was then added to the baseline model to assess model performance. The scaled Brier score was used to assess accuracy of prediction, scaled by its maximum score to range between 0 and 100% (Brier_scaled_ = 1 – Brier/Brier_max_, (where Brier_max_ = mean(*p*)*(1-*p*) and *p* = predicted probabilities), with higher values indicating better performance [39]. Discriminative ability was assessed using the concordance (c-) statistic. To improve estimates of the model performance statistics when performing internal validation, we used bootstrapping with 50 replications [40]. This was again replicated stratified by age (<75 and ≥75) to assess the validity in the younger and older populations.

The degree of overlap between the frailty measures and the routinely used Charlson Comorbidity Index (CCI) [41] was assessed. Given the interrelationship between co-morbidity and frailty, the diagnostic codes common to the CCI and each of the three frailty indices were identified. The number of common codes in the three frailty measures was, therefore, compared with those in the CCI.

Reliability was assessed by evaluating the consistency of the measures over time. The measures were calculated over a longer time period (2005–19) using the same inclusion criteria as stated before, allowing for assessment of any temporal changes in data. A descriptive analysis of the prevalence of frailty by year was undertaken by plotting the percentage of patients categorised annually as ‘fit’ (low-risk using HFRS, fit using SCARF or zero frailty syndromes using FS). To gauge expected changes in prevalence, we compared the trend in prevalence with that of the electronic Frailty Index (eFI) from published data [42].

All statistical analyses were undertaken in Stata 16.1.

## Results

### Study population

In total, 136,008 individuals diagnosed with CRC within the English NHS between 2016 and 2019 were included, with 36,255 (26.7%) dying within 1 year from the date of CRC diagnosis. The characteristics of this population are shown in Table 1.

**Table 1:** Characteristics of the study population

		All patients	Alive at 1-year	Death at 1-year
		*N* (%)	*N* (%)	*N* (%)
Median age (IQR)		73 (63–81)	71 (62–79)	79 (70–86)
Age (years)	18–64	37,476 (27.6)	32,132 (32.2)	5,342 (14.7)
	65–74	38,879 (28.6)	31,371 (31.5)	7,508 (20.7)
	75–84	39,715 (29.2)	27,064 (27.1)	12,651 (34.9)
	≥85	19,938 (14.7)	9,184 (9.2)	10,754 (29.7)
Sex	Male	75,999 (55.9)	57,244 (57.4)	18,755 (51.7)
	Female	60,009 (44.1)	42,509 (42.6)	17,500 (48.3)
Tumour site	Colon	99,077 (72.9)	69,614 (69.8)	29,463 (81.3)
	Rectum	36,931 (27.2)	30,139 (30.2)	6,792 (18.7)
Tumour stage	I	21,956 (16.1)	20,782 (20.8)	1,174 (3.2)
	II	31,085 (22.9)	27,849 (27.9)	3,236 (8.9)
	III	37,253 (27.4)	31,915 (32.0)	5,338 (14.7)
	IV	30,471 (22.4)	12,525 (12.6)	17,946 (49.5)
	Unknown	15,243 (11.2)	6,682 (6.7)	8,561 (23.6)
HFRS	Low risk	115,778 (85.1)	90,522 (90.8)	25,256 (69.7)
	Intermediate risk	15,020 (11.0)	7,586 (7.6)	7,434 (20.5)
	High risk	5,210 (3.8)	1,645 (1.7)	3,565 (9.8)
SCARF	Fit	75,328 (55.4)	60,810 (61.0)	14,518 (40.4)
	Mild frailty	26,012 (19.1)	19,185 (19.2)	6,827 (18.8)
	Moderate frailty	16,197 (11.9)	10,592 (10.6)	5,605 (15.5)
	Severe frailty	18,471 (13.6)	9,166 (9.2)	9,305 (25.7)
FS	0 Frailty syndromes	110,493 (81.2)	86,517 (86.7)	23,976 (81.2)
	1 Frailty syndrome	17,241 (12.7)	10,247 (10.3)	6,994 (19.3)
	2 Frailty syndromes	5,439 (4.0)	2,200 (2.2)	3,239 (8.9)
	≥3 Frailty syndromes	2,835 (2.1)	789 (0.8)	2,046 (5.6)
Total		136,008	99,753	36,255

### Feasibility

It was feasible to create all the measures. In total, the measures used 435 different ICD10 codes to quantify frailty: HFRS used 109, SCARF 377 and FS 49 codes (Supplementary Table 1). A total of 134,192 (98.7%) could be linked to a HES record in the 2 years prior to their CRC diagnosis. Of those who had linked HES data, just over half, 54.2% (*n* = 73,778), had no indication of frailty from any of the three measures.

### Validity

The prevalence of frailty varied across the three measures with 3.8% (*n* = 5210) of the study population classified as high-risk using the HFRS measure, 13.6% (*n* = 18,471) classified as having severe frailty using the SCARF measure and 2.1% (*n* = 2835) having ≥3 frailty syndromes using the FS model (Table 1). Across all three measures the proportion of individuals with a higher level of frailty at CRC diagnosis was greater amongst those who died within a year of their CRC diagnosis, compared with those who were alive at this point. Of those who died within a year of CRC diagnosis, 9.8% were classified as high risk using the HFRS, 25.7% as having severe frailty using SCARF and 5.6% as having ≥3 frailty syndromes using FS, compared with 1.7, 9.2 and 0.8% of those alive at 1-year (Table 1).

**Figure 1:**
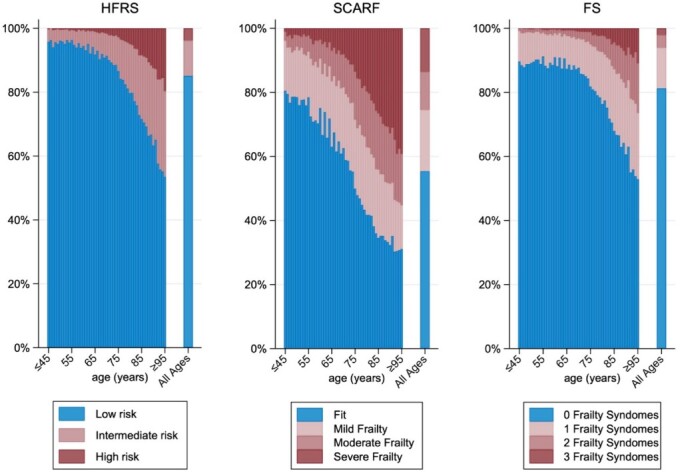
Prevalence of frailty by measure and age (years) in patients diagnosed with CRC in England, 2016–19.

Across all three measures, the prevalence of frailty increased with age. In HFRS, 0.6% of those aged <65 were classified as high risk and this increased to 11.4% amongst those aged ≥85. Using SCARF, the proportion classified as having severe frailty increased from 3.3% in those aged <65 to 30.9% in those aged ≥85. For FS, the proportion with ≥3 syndromes increased from 0.4% in the <65 age group to 6.4% in those aged ≥85 (Figure 1 and Supplementary Table 2). Using the SCARF index, the prevalence of frailty was higher in the younger age groups compared with the other measures; the SCARF index classified 74.4% of patients aged 18–64 as fit, compared with 94.8% as low-risk in the HFRS and 89.5% with 0 frailty syndromes. In all three measures, the proportion of individuals classified as fit (low risk HFRS, fit SCARF or 0 frailty syndromes) decreased rapidly from the age of 70 (Figure 1).

**Figure 2:**
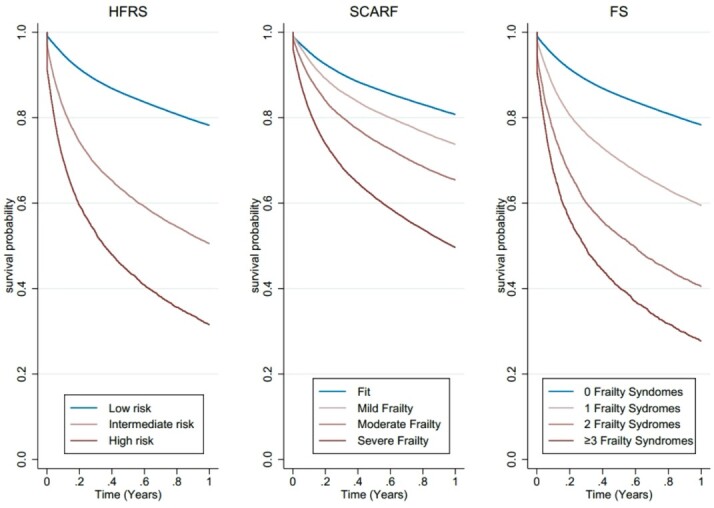
Category of frailty and 1-year overall survival across each measure.

The individuals classified as fit displayed comparable 1-year overall survival across the three measures (HFRS -78% (95%CI 78%–78%), SCARF—81% (95%CI 80%–81%), FS -78% (95%CI 78%–79%)) (Figure 2). HFRS and FS showed the greatest difference in 1-year overall survival between those with the lowest and highest levels of frailty, with a difference of >45% compared with 31% when using SCARF (Figure 2 and Table 2). Survival estimates were significantly different across categorical levels of frailty for all three measures (*P* < 0.01).

**Table 2:** One-year overall (%) survival by frailty measure and age (95% confidence interval)

**Frailty measure**		All patients	Aged <75 years	Aged ≥75 years
**HFRS**	Low risk	78 (78–78)	85 (85–85)	68 (67–68)
	Intermediate risk	50 (47–51)	63 (62–64)	45 (44–46)
	High risk	32 (31–33)	49 (45–52)	28 (26–29)
**SCARF**	Fit	81 (80–81)	87 (87–87)	68 (67–68)
	Mild frailty	74 (73–74)	80 (79–81)	67 (66–67)
	Moderate frailty	65 (65–66)	74 (73–75)	60 (59–61)
	Severe frailty	50 (49–50)	65 (64–67)	44 (44–45)
**FS**	0 Frailty syndromes	78 (78–79)	85 (51–86)	67 (67–68)
	1 Frailty syndrome	59 (59–60)	71 (70–72)	51 (50–52)
	2 Frailty syndromes	41 (39–41)	57 (55–60)	35 (33–36)
	≥3 Frailty syndromes	28 (26–29)	39 (35–43)	25 (23–27)

SCARF and HFRS had the largest overlap of codes, with 44 present in both, compared with 17 present in both SCARF and FS and none present in both HFRS and FS. Overall 20 codes were present in all three measures, with 302 codes only present in SCARF, 44 only in HFRS and 26 only in FS. All three frailty metrics had a degree of overlap with the CCI, the SCARF metric having the largest proportion of codes in common and FS having the least (Supplementary Table 3). A subset of the dementia component of the CCI was the only diagnoses present in all three frailty measures.

### Predictive validity

Both the c-statistic and scaled Brier score increased after adding a frailty measure to the baseline model showing an improvement in the prediction of 1-year overall mortality (Table 3). This was seen in the population as a whole and when stratified by age (<75 and ≥ 75). Overall, the model including the HFRS measure performed better than models including either SCARF or FS, but the differences were marginal (Table 3).

**Table 3:** c-statistic and scaled Brier score from logistic regression predicting 1-year mortality across the three frailty measures

	All patients		Aged <75 years		Aged ≥75 years	
	c	Brier	c	Brier	c	Brier
Baseline model^a^	0.849 (0.846–0.853)	32.7	0.837 (0.833–0.841)	25.3	0.820 (0.816–0.824)	31.4
+ HFRS	0.859 (0.857–0.862)	34.8	0.849 (0.845–0.851)	27.2	0.835 (0.830–0.839)	33.8
+ SCARF	0.857 (0.854–0.859)	34.1	0.851 (0.847–0.855)	27.1	0.830 (0.826–0.834)	32.8
+ FS	0.858 (0.857–0.862)	34.7	0.848 (0.843–0.851)	27.2	0.833 (0.828–0.837)	33.5

^a^Baseline model includes age, sex, tumour site and stage.

### Reliability

The percentage of patients categorised as fit decreased over time from 2005 to 2019 in the three measures (Supplementary Figure 1). Overall, the decrease was largest in SCARF (13.4 versus 8.8% in HFRS and 9.7% in FS). In all measures, larger decreases were observed in the older age groups and generally occurred in the early years of the study period. For comparison, published data on the eFI showed a decrease of 12.4% from 2006 to 2017, again with the largest decreases observed in the older age groups.

## Discussion

This study compared three different methods designed to quantify frailty using population-level administrative hospital data across a population of people diagnosed with CRC and has shown all have value. All were feasible to create, appeared to be valid, predicted short-term overall survival and were reliable. Each had different strengths and weaknesses which should be considered when deploying them in population-based epidemiological studies.

The prevalence of frailty differed substantially across the three measures. SCARF had the widest discrimination across the population with 55.4% of patients being deemed fit and 45.6% having some form of frailty. In contrast, HFRS and FS classified 85.1 and 81.2% in their lowest risk/frailty categories, respectively. This range in prevalence reflects the fact that frailty syndrome is a continuum whose severity is assessed on the basis of a constellation of conditions. The cut-off for the diagnosis of frailty using a particular score depends on the conditions assessed and the weight they are given.

Each of the measures was categorised differently with HFRS classifying patients into low, intermediate and high risk, SCARF into fit, mild, moderate or severe frailty and FS by number of frailty syndromes. This makes direct comparison across measures challenging, but may be relevant when considering which index to use in a particular analysis dependent of the research question. For example, FS may be preferable when specific frailty deficits are of interest in study.

The tools studied in this work were not initially developed for CRC patients. However, because conditions included are not specific to a single population, we believe in their values as frailty indicator to be used in population-based studies. This study was not intended to develop or adapt a frailty score for specific use in the CRC population, rather to test the feasibility of using existing scores in this population. The measures compared in this study are not designed for use in a clinical setting, rather to account for frailty in research using population-level data, contributing to hospital benchmarking and to inform public health interventions. The ability to quantify frailty outside of a clinical setting has implications for the management of CRC. Recent population-based studies have reported differing surgical treatment strategies in relation to age across multiple countries [13], and variation in the use of adjuvant chemotherapy in relation to age within the English NHS [14]. There is also evidence that cancer multidisciplinary team decisions are not implemented in a significant proportion of patients and patients being unfit for treatment may be responsible [43]. The addition of a frailty measure would allow for an assessment of fitness within these populations. Understanding the impact of frailty at a population level is part of working out why some patients do not receive what would seem to be the optimal treatment for their cancer.

A particular challenge when investigating frailty is distinguishing it from the related, but distinct, concept of co-morbidity. Comparisons of the codes used in the commonly used CCI with the frailty measures investigated in this study revealed considerable overlap for SCARF that would preclude using it alongside the CCI in an analysis, depending on the outcome in question SCARF or CCI could be utilised independently but the use of both together should be avoided. This reflects the challenges of frailty, and co-morbidity, assessment in epidemiological studies where the majority of perioperative clinical risk scores are based on the presence of absence or diagnostic codes [44, 45].

This challenge of quantifying frailty through diagnostic codes alone is, perhaps, the biggest limitation of all the frailty measures investigated. This study utilised diagnostic codes recorded during admissions in the 2-years preceding CRC diagnosis, therefore individuals who did not have an admission in that time period would be reported as not frail. Additionally, only the presence or absence of a condition was recorded so there was no indication of severity of condition. Frailty is a complex syndrome and reducing its quantification to the presence of absence of particular diagnoses in hospital attendances alone may be too simplistic. Variation in recording and coding of inpatient admissions over time may also introduce error into the measurement of individual components of frailty scores. For this reason, we limited the main analyses in this study to a 4-year period. In the extended analyses covering a longer time-period the measures displayed similar patterns of frailty prevalence to that of the eFI.

A further limitation of this study was that only measures constructed from secondary care data were considered. Stronger quantification of frailty would be expected from indices such as the eFI [46], or the preoperative frailty index [47] which include additional data sources such as primary care and prescription databases. Inevitably, measures captured through direct clinical assessment would provide the gold standard. This information is not routinely available to population-based epidemiological studies and, in their absence, HFRS, SCARF and FS provide a means to quantify frailty. Although that quantification can only ever be as complex and in depth as the data they are derived from, they still offer an important mechanism for considering its association with outcomes in descriptive epidemiological studies. The absence of a gold-standard measure of frailty to compare the measures used in this study can also be considered a limitation. However we compared each measure using an adverse outcome in the same way the measures were developed. The relationship with mortality is an important and non-arbitrary test of predictive validity [48].

HFRS, SCARF and FS already offer a mechanism for quantifying frailty and enabling its consideration in epidemiological studies. Given the growing evidence of persistent, and significant, age-related CRC inequities, and the uncertain contribution frailty may make in driving them, they are a valuable tool. Future studies should deploy them effectively to generate evidence that will help both identify, and eliminate, age-related inequities in CRC care and outcomes across the entire patient journey, and could seek to compare the association between frailty and treatment use and outcomes in different treatment pathways.

## Supplementary Material

Supplementary_materials_afae105

## References

[1] Fried LP , Ferrucci L, Darer J, Williamson JD, Anderson G. Untangling the concepts of disability, frailty, and comorbidity: implications for improved targeting and care. J Gerontol A Biol Sci Med Sci 2004; 59: 255–63. 15031310 10.1093/gerona/59.3.m255

[2] Fried LP , Tangen CM, Walston J et al. Frailty in older adults: evidence for a phenotype. J Gerontol A Biol Sci Med Sci 2001; 56: M146–57. 11253156 10.1093/gerona/56.3.m146

[3] Handforth C , Clegg A, Young C et al. The prevalence and outcomes of frailty in older cancer patients: a systematic review. Ann Oncol 2015; 26: 1091–101. 25403592 10.1093/annonc/mdu540

[4] Cancer Research UK . Cancer Incidence by Age 2022. Available at: https://www.cancerresearchuk.org/health-professional/cancer-statistics/incidence/age (23 November 2022, date last accessed).

[5] Richards SJ , Cherry TJ, Frizelle FA, Eglinton TW. Pre-operative frailty is predictive of adverse post-operative outcomes in colorectal cancer patients. ANZ J Surg 2021; 91: 379–86. 32975018 10.1111/ans.16319

[6] Ruiz J , Miller AA, Tooze JA et al. Frailty assessment predicts toxicity during first cycle chemotherapy for advanced lung cancer regardless of chronologic age. J Geriatr Oncol 2019; 10: 48–54. 30005982 10.1016/j.jgo.2018.06.007 PMC6785179

[7] Mima K , Miyanari N, Morito A et al. Frailty is an independent risk factor for recurrence and mortality following curative resection of stage I–III colorectal cancer. Ann Gastroenterol Surg 2020; 4: 405–12. 32724884 10.1002/ags3.12337 PMC7382441

[8] Cancer Research UK . Bowel Cancer Incidence by Age 2021. Available at: https://www.cancerresearchuk.org/health-professional/cancer-statistics/statistics-by-cancer-type/bowel-cancer/incidence#heading-One (2 March 2023, date last accessed).

[9] Macmillan Cancer Support . The Age Old Excuse: the Undertreatment of Older Cancer Patients 2012. Available at: https://www.macmillan.org.uk/documents/getinvolved/campaigns/ageoldexcuse/ageoldexcusereport-macmillancancersupport.pdf (16 October 2022, date last accessed).

[10] Birch RJ , Taylor JC, Downing A et al. Rectal cancer in old age–is it appropriately managed? Evidence from population-based analysis of routine data across the English national health service. Eur J Surg Oncol 2019; 45: 1196–204. 30661923 10.1016/j.ejso.2019.01.005 PMC6602152

[11] Taylor JC , Iversen LH, Burke D et al. Influence of age on surgical treatment and postoperative outcomes of patients with colorectal cancer in Denmark and Yorkshire, England. Colorectal Dis 2021; 23: 3152–61. 10.1111/codi.15910 34523211

[12] Michaud Maturana M , English WJ, Nandakumar M, Li Chen J, Dvorkin L. The impact of frailty on clinical outcomes in colorectal cancer surgery: a systematic literature review. ANZ J Surg 2021; 91: 2322–9. 34013571 10.1111/ans.16941

[13] Benitez Majano S , Di Girolamo C, Rachet B et al. Surgical treatment and survival from colorectal cancer in Denmark, England, Norway, and Sweden: a population-based study. Lancet Oncol 2019; 20: 74–87. 30545752 10.1016/S1470-2045(18)30646-6 PMC6318222

[14] Boyle JM , Kuryba A, Cowling TE et al. Determinants of variation in the use of adjuvant chemotherapy for stage III colon cancer in England. Clin Oncol 2020; 32: e135–44. 10.1016/j.clon.2019.12.008 31926818

[15] Williams GR , Al Obaidi M, Weaver A et al. Association between chronological age and geriatric assessment (GA) to identify deficits in elderly adults with cancer: findings from the CARE registry. J Clin Oncol 2020; 38: 12048.

[16] Quipourt V , Jooste V, Cottet V, Faivre J, Bouvier AM. Comorbidities alone do not explain the undertreatment of colorectal cancer in older adults: a French population-based study. J Am Geriatr Soc 2011; 59: 694–8. 21438864 10.1111/j.1532-5415.2011.03334.x

[17] Rockwood K , Song X, MacKnight C et al. A global clinical measure of fitness and frailty in elderly people. CMAJ 2005; 173: 489–95. 16129869 10.1503/cmaj.050051 PMC1188185

[18] Rolfson DB , Majumdar SR, Tsuyuki RT, Tahir A, Rockwood K. Validity and reliability of the Edmonton Frail Scale. Age Ageing 2006; 35: 526–9. 16757522 10.1093/ageing/afl041 PMC5955195

[19] Morley JE , Malmstrom TK, Miller DK. A simple frailty questionnaire (FRAIL) predicts outcomes in middle aged African Americans. J Nutr Health Aging 2012; 16: 601–8. 22836700 10.1007/s12603-012-0084-2 PMC4515112

[20] Pilotto A , Ferrucci L, Franceschi M et al. Development and validation of a multidimensional prognostic index for one-year mortality from comprehensive geriatric assessment in hospitalized older patients. Rejuvenation Res 2008; 11: 151–61. 18173367 10.1089/rej.2007.0569 PMC2668166

[21] Gobbens RJ , van Assen MA, Luijkx KG, Wijnen-Sponselee MT, Schols JM. The Tilburg Frailty Indicator: psychometric properties. J Am Med Dir Assoc 2010; 11: 344–55. 20511102 10.1016/j.jamda.2009.11.003

[22] Peters LL , Boter H, Buskens E, Slaets JP. Measurement properties of the Groningen Frailty Indicator in home-dwelling and institutionalized elderly people. J Am Med Dir Assoc 2012; 13: 546–51. 22579590 10.1016/j.jamda.2012.04.007

[23] Subra J , Gillette-GuyonnetS, CesariMet al. The integration of frailty into clinical practice: preliminary results from the Gerontopole. J Nutr Health Aging2012; 16: 714–20.23076514 10.1007/s12603-012-0391-7

[24] Gilbert T , Neuburger J, Kraindler J et al. Development and validation of a Hospital Frailty Risk Score focusing on older people in acute care settings using electronic hospital records: an observational study. Lancet (London, England) 2018; 391: 1775–82. 29706364 10.1016/S0140-6736(18)30668-8 PMC5946808

[25] Jauhari Y , Gannon MR, Dodwell D et al. Construction of the secondary care administrative records frailty (SCARF) index and validation on older women with operable invasive breast cancer in England and Wales: a cohort study. BMJ Open 2020; 10: e035395. 10.1136/bmjopen-2019-035395 PMC7223146 32376755

[26] Soong J , Poots AJ, Scott S, Donald K, Bell D. Developing and validating a risk prediction model for acute care based on frailty syndromes. BMJ Open 2015; 5: e008457. 10.1136/bmjopen-2015-008457 PMC4621379 26490098

[27] World Health Organization . ICD 10: International Statistical Classification of Diseases and Related Health Problems. 10th edition. Washington: Amer Psychiatric Pub, 1992.

[28] NHS Digital . Hospital Episode Statistics (HES) 2022. Available at: https://digital.nhs.uk/data-and-information/data-tools-and-services/data-services/hospital-episode-statistics (17 October 2022, date last accessed).

[29] Downing A , Hall P, Birch R et al. Data resource profile: the COloRECTal cancer data repository (CORECT-R). Int J Epidemiol 2021; 50: 1418–k. 34255059 10.1093/ije/dyab122 PMC8580263

[30] Soong J , Poots AJ, Scott S et al. Quantifying the prevalence of frailty in English hospitals. BMJ Open 2015; 5: e008456. 10.1136/bmjopen-2015-008456 PMC4621378 26490097

[31] Steptoe A , Breeze E, Banks J, Nazroo J. Cohort profile: the English longitudinal study of ageing. Int J Epidemiol 2013; 42: 1640–8. 23143611 10.1093/ije/dys168 PMC3900867

[32] Lüchtenborg M , Morris EJA, Tataru D et al. Investigation of the international comparability of population-based routine hospital data set derived comorbidity scores for patients with lung cancer. Thorax 2018; 73: 339–49. 29079609 10.1136/thoraxjnl-2017-210362 PMC5870453

[33] Hendifar A , Yang D, Lenz F et al. Gender disparities in metastatic colorectal cancer survival. Clin Cancer Res 2009; 15: 6391–7. 19789331 10.1158/1078-0432.CCR-09-0877 PMC2779768

[34] Yang Y , Wang G, He J et al. Gender differences in colorectal cancer survival: a meta-analysis. Int J Cancer 2017; 141: 1942–9. 28599355 10.1002/ijc.30827

[35] Quaglia A , Tavilla A, Shack L et al. The cancer survival gap between elderly and middle-aged patients in Europe is widening. Eur J Cancer 2009; 45: 1006–16. 19121578 10.1016/j.ejca.2008.11.028

[36] van Eeghen EE , Bakker SD, van Bochove A, Loffeld RJ. Impact of age and comorbidity on survival in colorectal cancer. J Gastrointest Oncol 2015; 6: 605–12. 26697191 10.3978/j.issn.2078-6891.2015.070 PMC4671847

[37] Maringe C , Walters S, Rachet B et al. Stage at diagnosis and colorectal cancer survival in six high-income countries: a population-based study of patients diagnosed during 2000–2007. Acta Oncol 2013; 52: 919–32. 23581611 10.3109/0284186X.2013.764008

[38] Lee Y-C , Lee Y-L, Chuang J-P, Lee J-C. Differences in survival between colon and rectal cancer from SEER data. PloS One 2013; 8: e78709. 24265711 10.1371/journal.pone.0078709 PMC3827090

[39] Steyerberg EW , Harrell FE Jr, Borsboom GJ, Eijkemans MJ, Vergouwe Y, Habbema JD. Internal validation of predictive models: efficiency of some procedures for logistic regression analysis. J Clin Epidemiol 2001; 54: 774–81. 11470385 10.1016/s0895-4356(01)00341-9

[40] Fernandez-Felix BM , García-Esquinas E, Muriel A, Royuela A, Zamora J. Bootstrap internal validation command for predictive logistic regression models. Stata J 2021; 21: 498–509.

[41] Charlson ME , Pompei P, Ales KL, MacKenzie CR. A new method of classifying prognostic comorbidity in longitudinal studies: development and validation. J Chronic Dis 1987; 40: 373–83. 3558716 10.1016/0021-9681(87)90171-8

[42] Walsh B , Fogg C, Harris S et al. Frailty transitions and prevalence in an ageing population: longitudinal analysis of primary care data from an open cohort of adults aged 50 and over in England, 2006-2017. Age Ageing 2023; 52: afad058. 10.1093/ageing/afad058 PMC10158172 37140052

[43] Blazeby JM , Wilson L, Metcalfe C, Nicklin J, English R, Donovan JL. Analysis of clinical decision-making in multi-disciplinary cancer teams. Ann Oncol 2006; 17: 457–60. 16322114 10.1093/annonc/mdj102

[44] Raval MV , Pawlik TM. Practical guide to surgical data sets: national surgical quality improvement program (NSQIP) and pediatric NSQIP. JAMA Surg 2018; 153: 764–5. 29617521 10.1001/jamasurg.2018.0486

[45] Copeland G , Jones D, Walters M. POSSUM: a scoring system for surgical audit. Br J Surg 1991; 78: 355–60. 2021856 10.1002/bjs.1800780327

[46] Clegg A , Bates C, Young J et al. Development and validation of an electronic frailty index using routine primary care electronic health record data. Age Ageing 2016; 45: 353–60. 26944937 10.1093/ageing/afw039 PMC4846793

[47] McIsaac DI , Wong CA, Huang A, Moloo H, van Walraven C. Derivation and validation of a generalizable preoperative frailty index using population-based health administrative data. Ann Surg 2019; 270: 102–8. 29672410 10.1097/SLA.0000000000002769

[48] Searle SD , Mitnitski A, Gahbauer EA, Gill TM, Rockwood K. A standard procedure for creating a frailty index. BMC Geriatr 2008; 8: 24. 18826625 10.1186/1471-2318-8-24 PMC2573877

