# Supplementary material for: A comparison of frailty measures in population-based data for patients with colorectal cancer
Source: Age Ageing. 2024 May 23;53(5):afae105. doi: 10.1093/ageing/afae105 (PMC11116828; doi:10.1093/ageing/afae105)
Supplement: Supplementary_materials_afae105 [file supplementary_materials_afae105.docx]

**SUPPLEMENTARY TABLES & FIGURES**

**Supplementary figure 1: Percentage of patients with colorectal cancer classified as fit in the HFRS, SCARF and FS measures by age group from 2005 to 2019. The percentage of general population patients classified as fit by the eFI in Walsh et al(39) from 2006 to 2017 are also given for reference. Age groups were used to match those in Walsh et al (39).**

**
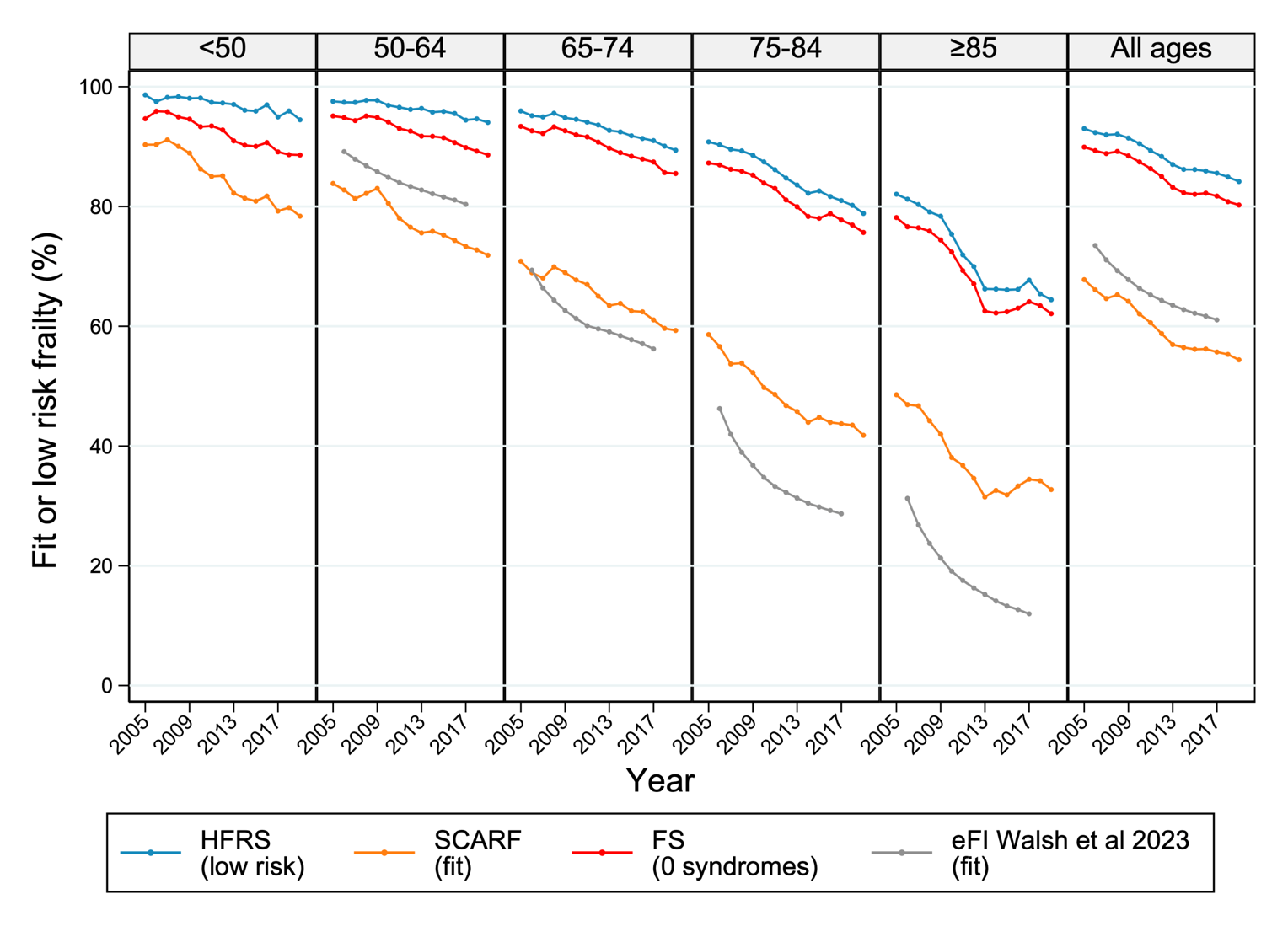
**

**Supplementary table 1: ICD10 codes used to identify frailty for each measure**

| **ICD10 code category** | **ICD10 codes** |
| --- | --- |
| SCARF | |
| Certain infectious and parasitic diseases (A00-B99) | B353 |
| Diseases of the blood and blood-forming organs (D50-D89) | D50, D51, D52, D53, D63, D64 |
| Endocrine, nutritional and metabolic diseases (E00-E90) | E03, E04, E05, E06, E079, E10-E14, E41, E43, E44, E46, E53, E55, E66, E83, E86, E87 |
| Mental and behavioural disorders (F00-F99) | F00, F01, F02, F03, F04, F05, F067, F10, F11, F12, F13, F14, F15, F16, F17, F18, F19, F20, F21, F22, F23, F24, F25, F28, F29, F30, F31, F32, F33, F34, F38, F39, F41 |
| Diseases of the nervous system (G00-G99) | G11, G122, G20, G21, G22, G23, G25, G26, G30, G31, G32, G35, G45, G46, G575, G576, G590, G632, G81, G82, G83 |
| Diseases of the eye and adnexa (H00-H59) | H25, H28, H35, H360, H40, H43, H53, H54 |
| Diseases of the ear and mastoid process (H60-H95) | H833, H90, H91 |
| Diseases of the circulatory system (I00-I99) | I05, I06, I07, I08, I10, I11, I12, I13, I20, I21, I22, I23, I24, I250, I251, I252, I253, I254, I255 , I256, I258, I259, I260, I27, I34, I35, I36, I37, I390, I391, I392, I393, I394, I42, I43, I44, I48, I49, I50, I51, I60, I61, I62, I63, I64, I65, I66, I67, I68, I69, I70, I71, I72, I73, I770, I771, I83, I95, I98 |
| Diseases of the respiratory system (J00-J99) | J13, J14, J15, J16, J18, J20, J22, J40, J41, J42, J43, J44, J45, J46,  J47, J60, J61, J62, J63, J64, J65, J684, J70, J90, J961, J98 |
| Diseases of the digestive system (K00-K93) | K21, K25, K26, K27, K29, K551, K558, K559 |
| Diseases of the skin and subcutaneous tissue (L00-L99) | L03, L08, L60, L89, L97, L984 |
| Diseases of the musculoskeletal system and connective tissue (M00-M99) | M05, M06, M07, M09, M10, M11, M12, M13, M142, M146, M15, M16, M17, M18, M19, M201, M202, M203, M204, M205, M206, M213, M214, M215, M216, M315, M32, M33, M34, M35, M36, M484, M62, M722, M766, M773, M775, M80, M81, M82 |
| Diseases of the genitourinary system (N00-N99) | N01, N03, N05, N07, N08, N083, N18, N19, N25, N30, N31, N34, N390, N393, N394, N398, N399 |
| Congenital malformations, deformations and chromosomal abnormalities (Q00-Q99) | Q66 |
| Symptoms, signs and abnormal clinical and laboratory findings, not elsewhere classified (R00-R99) | R02, R06, R12, R15, R25, R26, R296, R31, R32, R33, R40, R41, R42, R45, R460, R468, R53, R54, R55, R628, R63, R64 |
| Injury, poisoning and certain other consequences of external causes (S00-T98) | S00, S01, S22, S32, S33, S42, S43, S62, S72, S73, S78, S88, S90, S91, S92, S93, S94, S95, S96, S97, S98, S99, T136, T835 |
| External causes of morbidity and mortality (V01-Y98) | W00, W01, W04, W05, W06, W07, W08, W10, W18, W19, X53, Y06, Y83, Y84 |
| Factors influencing health status and contact with health services (Z00-Z99) | Z450, Z453, Z461, Z466, Z49, Z50, Z59, Z60, Z63, Z73, Z74, Z755, Z940, Z950, Z952, Z953, Z954, Z958, Z959, Z974, Z992, Z993, Z998, Z999 |
| HFRS | |
| Certain infectious and parasitic diseases (A00-B99) | A04, A09, A41, B95, B96 |
| Diseases of the blood and blood-forming organs (D50-D89) | D64 |
| Endocrine, nutritional and metabolic diseases (E00-E90) | E05, E16, E53, E55, E83, E86, E87 |
| Mental and behavioural disorders (F00-F99) | F00, F01, F03, F05, F10, F32 |
| Diseases of the nervous system (G00-G99) | G20, G30, G31, G40, G45, G81 |
| Diseases of the eye and adnexa (H00-H59) | H54 |
| Diseases of the ear and mastoid process (H60-H95) | H91 |
| Diseases of the circulatory system (I00-I99) | I63, I67, I69, I95 |
| Diseases of the respiratory system (J00-J99) | J18, J22, J69, J96 |
| Diseases of the digestive system (K00-K93) | K26, K52, K59, K92 |
| Diseases of the skin and subcutaneous tissue (L00-L99) | L03, L08, L89, L97 |
| Diseases of the musculoskeletal system and connective tissue (M00-M99) | M15, M19, M25, M41, M48, M79, M80, M81 |
| Diseases of the genitourinary system (N00-N99) | N17, N18, N19, N20, N28, N39 |
| Symptoms, signs and abnormal clinical and laboratory findings, not elsewhere classified (R00-R99) | R00, R02, R11, R13, R26, R29, R31, R32, R33, R40, R41, R44, R45, R47, R50, R54, R55, R56, R63, R69, R79, R94 |
| Injury, poisoning and certain other consequences of external causes (S00-T98) | S00, S01, S06, S09, S22, S32, S42, S51, S72, S80, T83 |
| External causes of morbidity and mortality (V01-Y98) | W01, W06, W10, W18, W19, X59, Y95 |
| Factors influencing health status and contact with health services (Z00-Z99) | Z22, Z50, Z60, Z73, Z74, Z75, Z87, Z91, Z99 |
| Codes for special purposes (U00-U99) | U80 |
| FS | |
| Mental and behavioural disorders (F00-F99) | F00, F01, F02, F03, F04, F05, F32, F33, F38, F41, F43, F44 |
| Diseases of the skin and subcutaneous tissue (L00-L99) | L89 |
| Symptoms, signs and abnormal clinical and laboratory findings, not elsewhere classified (R00-R99) | R15, R26, R32, R41, R54, R55 |
| Injury, poisoning and certain other consequences of external causes (S00-T98) | S32, S33, S42, S43, S62, S72, S73 |
| External causes of morbidity and mortality (V01-Y98) | W00, W01, W02, W03, W04, W05, W06, W07, W08, W09, W10, W11, W12, W13, W14, W15, W16, W17, W18, W19 |
| Factors influencing health status and contact with health services (Z00-Z99) | Z74, Z75, Z93 |

**Supplementary table 2: Prevalence (%) of frailty by age group.**

|  |  | Age at cancer diagnosis (years) | | | |
| --- | --- | --- | --- | --- | --- |
| **Frailty measure and level** |  | 18-64 | 65-74 | 75-84 | ≥85 |
| **HFRS** | Low risk | 94.8 | 90.4 | 80.4 | 65.9 |
|  | Intermediate risk | 4.5 | 7.7 | 14.6 | 22.7 |
|  | High risk | 0.6 | 1.8 | 5.0 | 11.4 |
| **SCARF** | Fit | 74.4 | 60.6 | 43.2 | 33.7 |
|  | Mild frailty | 16.3 | 20.0 | 21.3 | 18.4 |
|  | Moderate frailty | 6.0 | 10.7 | 16.2 | 17.0 |
|  | Severe frailty | 3.3 | 8.8 | 19.3 | 30.9 |
| **Frailty Syndromes** | 0 Frailty syndromes | 89.5 | 86.6 | 77.3 | 63.2 |
|  | 1 Frailty syndrome | 8.9 | 10.1 | 15.1 | 20.1 |
|  | 2 Frailty Syndromes | 1.2 | 2.4 | 5.1 | 10.3 |
|  | ≥3 Frailty syndromes | 0.4 | 0.9 | 2.6 | 6.4 |

**Supplementary table 3: ICD10 codes included in the Charlson comorbidity score, and associated frailty indices**

| **Charlson Component** | **ICD10-Codes** | **Present in Frailty Measure** | | |
| --- | --- | --- | --- | --- |
|  |  | **SCARF** | **HFRS** | **FS** |
| Cerebrovascular Disease | I60, I61, I62, I64, I65, I66, I68, G46 | Yes | No | No |
|  | I63, I67, I69, G45 | Yes | Yes | No |
| Pulmonary disease | J40, J41, J42, J43, J44, J45, J46, J47, J60, J61, J62, J63, J64, J65, I278, I279, J684, J701, J703 | Yes | No | No |
|  | J66, J67 | No | No | No |
| Congestive heart failure | I43, I50, I110, I130, I132, I255, I420, I425, I426, I427, I428, I429 | Yes | No | No |
|  | I099, P290 | No | No | No |
| Dementia | F00, F01, F03, F051 | Yes | Yes | Yes |
|  | F02 | Yes | No | Yes |
|  | G30, G311 | Yes | Yes | No |
| Myocardial infarction | I21, I22, I252 | Yes | No | No |
| Peptic ulcer | K25, K27 | Yes | No | No |
|  | K26 | Yes | Yes | No |
|  | K28 | No | No | No |
| Peripheral vascular disease | I70, I71, I731, I738, I739, I771, K551, K558, K559, Z958 | Yes | No | No |
|  | I790, I792 | No | No | No |
| Rheumatic disease | M05, M06, M32, M33, M34, M315, M351, M353, M360 | Yes | No | No |
| Mild liver disease | K73, K74, K702, K703, K760, K762, K763, K764, K768, K769, Z944 | No | No | No |
| Diabetes without complications | E100, E101, E106, E108, E109, E110, E111, E116, E118, E119, E120, E121, E126, E128, E129, E130, E131, E136, E138, E139, E140, E141, E146, E148, E149 | Yes | No | No |
| Diabetes with complications | E102, E103, E104, E105, E107, E112, E113, E114, E115, E117, E122, E123, E124, E125, E127, E132, E133, E134, E135, E137, E142, E143, E144, E145, E147 | Yes | No | No |
| Cancer | C00 to C26, C30 to C34, C37 to C41, C43, C45, C58, C60 to C76, C81 to C85, C88, C90 to C97 | No | No | No |
| Hemiplegia or paraplegia | G041, G801, G802 | No | No | No |
|  | G114, G82, G830, G831, G832, G833, G834, G839 | Yes | No | No |
|  | G81 | Yes | Yes | No |
| Renal disease | I12, I130, I131, I132, N032, N033, N034, N035, N036, N037, N052, N053, N054, N055, N056, N057, N250, Z491, Z492, Z940 | Yes | No | No |
|  | N18, N19, Z992 | Yes | Yes | No |
| Severe liver disease | I850, I859, I864, K704, K721, K729, K765, K766, K767 | No | No | No |
|  | I982 | Yes | No | No |
| Metastatic cancer | C77, C78, C79, C80 | No | No | No |
| HIV | B20, B21, B22, B24 | No | No | No |
